# Association between *MTNR1B* polymorphisms and obesity in African American: findings from the Jackson Heart Study

**DOI:** 10.1186/s12920-021-00983-2

**Published:** 2021-05-21

**Authors:** Cynthia Tchio, Solomon K. Musani, Alexander Quarshie, Gianluca Tosini

**Affiliations:** 1 Circadian Rhythms and Sleep Disorders Program, Neuroscience Institute, Morehouse School of Medicine, 720 Westview Dr. SW, Atlanta, GA 30130 USA; 2 Department of Pharmacology and Toxicology, Morehouse School of Medicine, Atlanta, GA USA; 3 Jackson Heart Study, University of Mississippi Medical Center, Jackson, MS USA; 4 Clinical Research Center, Morehouse School of Medicine, Atlanta, GA USA

**Keywords:** *MTNR1B*, Obesity, Body mass index, Waist circumference, Adiposity, Insomnia, African american

## Abstract

**Background:**

Melatonin is a hormone that is secreted at night by the pineal gland. It exerts its function by binding to the MT_1_ and MT_2_ receptors, which are encoded by the *MTNR1A* and *MTNR1B* genes, respectively. Previous studies reveal that *MTNR1B* variants are associated with insulin secretion impairments and an increased body mass index (BMI) in individuals of European and Asian ancestries. Obesity is highly prevalent in the US and disproportionately affects African Americans. Here, we hypothesized that common single nucleotide polymorphisms (SNPs) imputed in 1000 Genomes in the *MTNR1B* gene are associated with adiposity in African American adult men and women and that the association is modified by insomnia.

**Methods:**

We used an additive genetic model to describe the association between the adiposity traits (BMI and waist circumference) and selected *MTNR1B* variants in 3,029 Jackson Heart Study participants, with an average age of 55.13 ± 12.84 years, and 62% were women. We regressed the adiposity measures on the estimated allelic or genotypic dosage at every selected SNP and adjusted for age, sex, population stratification, and insomnia. Thirty common SNPs, spanning the *MTNR1B* gene, with a minor allele frequency ≥ 5%, a call rate ≥ 90%, a Hardy–Weinberg equilibrium *p* value > 10^–6^, were available for the analysis.

**Results:**

The allele T of rs76371840 was associated with adiposity (OR = 1.47 [1.13—1.82]; P_FDR-adjusted_ = 0.0499), and the allele A of rs8192552 showed a significant association with waist circumference (β = 0.023 ± 0.007; P_FDR-adjusted_ = 0.0077) after correcting for multiple testing. When insomnia was included in the adiposity analysis model, the following four variants became significantly associated with adiposity: rs6483208; rs4388843; rs4601728; and rs12804291.

**Conclusions:**

Our data indicate that polymorphisms in the *MTNR1B* gene are associated with obesity traits in African Americans. To the best of our knowledge, this is the first study to explore the effect of insomnia on the association between the circadian *MTNR1B* genetic variants and metabolic traits in an African American sample population. We observed that insomnia affected the association between the *MTNR1B* variants and adiposity.

## Background

Melatonin is a chronobiotic hormone that is synthesized by the pineal gland at night [1]. Melatonin is not stored within the pineal gland, but due to its lipophilic nature, it diffuses in the bloodstream, where it rapidly reaches target tissues. Melatonin’s function is exerted in a tissue-specific manner by binding to specific G-coupled protein receptors known as melatonin receptor type 1 (MT_1_) and melatonin receptor type 2 (MT_2_). These receptors are present in many tissues and organs [1, 2]. In humans, MT_1_ and MT_2_ are encoded by the *MTNR1A* and *MTNR1B* genes, respectively.

Multiple studies demonstrate that melatonin is involved in regulating sleep, circadian rhythms, reproduction, and metabolic processes [3–5]. Polymorphisms in the *MTNR1B* gene have been linked to impairments in insulin secretion, fasting blood glucose (FBG) levels, and an increased body mass index (BMI) in individuals of European and Asian ancestry [6–9]. There are various potential mechanisms by which polymorphisms in the *MTNR1B* gene might increase FBG levels. It has been reported that the common variant rs10830963, which increases MT_2_ signaling, might also be associated with an increased risk of developing type 2 diabetes (T2D) [9, 10]. Other studies in individuals of European ancestry show that rare variants in the *MTNR1B* gene result in reduced or absent MT_2_ signaling, which is also associated with an increased FBG level and an increased risk of developing T2D [11–13].

Obesity is defined as a BMI greater than 30, and it is a growing pandemic that affects 36.5% of US adults [14]. Obesity is prevalent across different ethnic groups, with African Americans having the highest age-adjusted rates [15]. Although African Americans have a higher prevalence of obesity [15] and the highest prevalence of short sleep duration and insomnia compared to other ethnicities [16–18], there are no documented studies on the influence of *MTNR1B* on adiposity or the effect of insomnia on this association. This study aimed to investigate whether selected common genetic variations in the *MTNR1B* gene reported in previous studies were associated with adiposity in a sample of African American individuals and whether those associations were modified by insomnia.

## Results

### Study population characteristics

The study population characteristics are displayed in Table 1. Of the available study sample, the average age was 55 years old, and 1871 (62%) were women. The participants had a mean BMI of 32 kg/m^2^, a mean waist circumference of 101 cm**,** and a total cholesterol of 198 mg/dL. Also, 679 (29%) participants had diabetes, and the study population had a mean fasting glucose of 100 mg/dL, a mean HbA1c percentage of 6, and a fasting insulin of 19 IU/mL. Additionally, 98 (5%) participants reported insomnia.

**Table 1:** Clinical characteristics of the study population

Participants (male /female)	3029 (1149/1871)
Age (years)	55.13 ± 12.84
Adiposity (yes/no)	2450/578
BMI (kg/m^2^)	32.02 ± 7.44
Waist circumference (cm)	101.34 ± 16.31
Total cholesterol (mg/dL)	198.42 ± 40.59
LDL (mg/dL)	126.6 ± 36.6
HDL (mg/dL)	51.25 ± 14.67
Type 2 diabetes (Yes/No)	679/2336
Fasting plasma glucose (mg/dL)	100.54 ± 33.71
Fasting insulin (IU/mL)	18.75 ± 24.25
HOMA IR	3.62 ± 2.32
HbA1c (%)	6.00 ± 1.33
Nighttime sleep (h)	6.43 ± 1.52
Insomnia (yes/no)	98/1976

### The ASSOCIATION of *MTNR1B* SNPs with BMI and waist circumference

As shown in Table 2, multiple linear regression models identified sixteen *MTNR1B* variants that showed a nominally significant association (FDR *p* value ≤ 0.05) with continuous obesity traits (BMI and waist circumference). Fourteen *MTNR1B* SNPs were inversely and significantly associated with BMI. Ten of these remained significant after adjusting for multiple testing. For waist circumference, sixteen *MTNR1B* SNPs showed a significant inverse association, while rs8192552 showed a significant direct effect.

**Table 2:** The association of *MTNR1B* SNPs with BMI and waist circumference

					BMI				Waist circumference				
SNPs	BP	A1	A2	MAF	*p* value	FDR * p* value	Beta	SE (Beta)	*p* value	FDR * p* value	Beta	SE (Beta)	Type of Mutation
rs3781638	92980341	T	G	0.499	0.0041	0.0200	−0.0158	0.0055	0.0006	0.0075	−0.0140	0.0041	Intronic
rs1562444	92982683	A	G	0.446	0.0056	0.0200	−0.0153	0.0055	0.0007	0.0075	−0.0138	0.0041	3'-UTR
rs12792653	92982750	A	G	0.446	0.0056	0.0200	−0.0153	0.0055	0.0008	0.0075	−0.0138	0.0041	3'-UTR
rs8192552	92969796	G	A	0.094	0.0432	0.0925	0.0189	0.0094	0.0010	0.0077	0.0228	0.0069	Missense
rs6483209	92976223	C	T	0.212	0.0025	0.0200	−0.0201	0.0067	0.0020	0.0089	−0.0153	0.0050	Intronic
rs12290860	92979012	G	A	0.224	0.0060	0.0200	−0.0184	0.0067	0.0017	0.0089	−0.0155	0.0049	Intronic
rs7127128	92980510	A	G	0.227	0.0029	0.0200	−0.0196	0.0066	0.0024	0.0089	−0.0149	0.0049	Intronic
rs6483210	92981429	C	T	0.227	0.0029	0.0200	−0.0197	0.0066	0.0025	0.0089	−0.0148	0.0049	Intronic
rs61747139	92981951	A	G	0.227	0.0030	0.0200	−0.0196	0.0066	0.0027	0.0089	−0.0147	0.0049	Missense
rs10765576	92973778	G	A	0.489	0.0332	0.0831	−0.0117	0.0055	0.0038	0.0096	−0.0117	0.0040	Intronic
rs11020127	92974394	C	A	0.495	0.0050	0.0200	−0.0186	0.0067	0.0034	0.0096	−0.0119	0.0041	Intronic
rs12292400	92979446	G	C	0.225	0.0394	0.0909	−0.0112	0.0054	0.0038	0.0096	−0.0143	0.0049	Intronic
rs12272268	92975633	C	G	0.252	0.0184	0.0501	−0.0147	0.0063	0.0048	0.0111	−0.0131	0.0046	Intronic
rs7130424	92980527	T	C	0.266	0.0099	0.0298	−0.0160	0.0062	0.0054	0.0116	−0.0128	0.0046	Intronic
rs11020126	92973751	G	A	0.181	0.0606	0.1212	−0.0136	0.0072	0.0165	0.0331	−0.0129	0.0054	Intronic
rs11020125	92973378	T	G	0.171	0.0895	0.1679	−0.0124	0.0073	0.0218	0.0409	−0.0124	0.0054	Intronic
rs6483208	92972678	T	G	0.22	0.1446	0.2411	−0.0096	0.0066	0.0287	0.0507	−0.0107	0.0049	Intronic

BMI for adjusted age, sex, and ancestry; Waist Circumference for adjusted age, sex, and ancestry. The associations' results are Beta, standard error (SE), and the corresponding adjusted False Discovery Rate (FDR) *p* value. A1 and A2 are reference allele and alternative allele; MAF is Minor Allele Frequency; BP is Base Pair

### Association between *MTNR1B* variants with adiposity adjusting for insomnia

A multiple logistic regression model identified sixteen *MTNR1B* variants with a significant association with adiposity (Table 3). After adjusting for insomnia, four new variants, not identified in our previous models, showed a nominally significant association (FDR *p* value ≤ 0.05) with adiposity (Table 3). Based on the functional annotation of the *MTNR1B* variants, only two of the variations were missense variants, and we identified variants in other regulatory regions. In the regulatory regions of the *MTNR1B* introns, two of the variants were in the 3 prime-untranslated region (3’-UTR).

**Table 3:** The Association between MTNR1B Variants and Adiposity adjusting for Insomnia

					Adiposity					Adiposity-Insomnia					
SNPs	BP	A1	A2	MAF	*p* value	FDR *p* value	OR	LCI	UCI	*p* value	FDR *p* value	OR	LCI	UCI	Type of Mutation
rs12290860	92979012	G	A	0.2240	0.0042	0.0250	0.74	0.54	0.95	0.0012	0.0040	0.66	0.40	0.91	Intronic
rs61747139	92981951	A	G	0.2270	0.0042	0.0250	0.74	0.54	0.95	0.0013	0.0040	0.66	0.41	0.92	Missense
rs6483210	92981429	C	T	0.2270	0.0043	0.0250	0.75	0.54	0.95	0.0013	0.0040	0.66	0.41	0.92	Intronic
rs7127128	92980510	A	G	0.2270	0.0044	0.0250	0.75	0.54	0.95	0.0013	0.0040	0.66	0.41	0.92	Intronic
rs12292400	92979446	G	C	0.2250	0.0055	0.0250	0.75	0.55	0.95	0.0017	0.0047	0.67	0.41	0.92	Intronic
rs6483209	92976223	C	T	0.2120	0.0022	0.0250	0.73	0.52	0.93	0.0003	0.0025	0.62	0.36	0.88	Intronic
rs7130424	92980527	T	C	0.2660	0.0117	0.0281	0.79	0.60	0.97	0.0021	0.0053	0.70	0.46	0.93	Intronic
rs12272268	92975633	C	G	0.2520	0.0075	0.0250	0.77	0.58	0.96	0.0007	0.0040	0.67	0.43	0.91	Intronic
rs12792653	92982750	A	G	0.4460	0.0121	0.0281	0.81	0.64	0.97	0.0872	0.1313	0.83	0.62	1.04	3’-UTR
rs1562444	92982683	A	G	0.4460	0.0122	0.0281	0.81	0.64	0.98	0.0875	0.1313	0.83	0.62	1.04	3’-UTR
rs11020126	92973751	G	A	0.1810	0.0060	0.0250	0.73	0.51	0.96	0.000045	0.0012	0.56	0.27	0.84	Intronic
rs11020125	92973378	T	G	0.1710	0.0070	0.0250	0.74	0.51	0.96	0.0001	0.0012	0.57	0.28	0.86	Intronic
rs12277904	92978680	C	T	0.1370	0.0105	0.0281	0.73	0.48	0.97	0.0115	0.0203	0.68	0.38	0.98	Intronic
rs116625623	92971729	G	T	0.0950	0.0180	0.0385	0.70	0.41	1.00	0.0012	0.0040	0.54	0.17	0.92	Intronic
rs7129768	92980510	G	A	0.0880	0.0202	0.0404	0.70	0.40	1.00	0.0033	0.0075	0.56	0.18	0.95	Intronic
rs76371840	92971529	C	T	0.0740	0.0266	0.0499	1.48	1.13	1.82	0.0423	0.0705	1.54	1.12	1.96	Intronic
rs6483208	92972678	T	G	0.2200	0.0291	0.0514	0.80	0.60	1.00	0.0003	0.0025	0.63	0.38	0.89	Intronic
rs4388843	92971820	G	A	0.3110	0.2311	0.3015	0.90	0.72	1.08	0.0058	0.0123	0.73	0.50	0.95	Intronic
rs4601728	92971992	A	G	0.3130	0.2432	0.3040	0.90	0.72	1.08	0.0066	0.0132	0.73	0.51	0.96	Intronic
rs12804291	92972141	C	T	0.2650	0.2203	0.3005	0.89	0.71	1.08	0.0092	0.0172	0.73	0.50	0.97	Intronic

Adiposity for adjusted age, sex, and ancestry; Adiposity-Insomnia for Adiposity model with adjustment for Insomnia. The results of the associations are listed as Odds Ratio (OR) with the Lower and Upper Confidence Interval (LCI and UCI) and the corresponding adjusted False Discovery Rate (FDR) *p* value. A1 and A2 are reference allele and alternative allele; MAF is Minor Allele Frequency; BP is Base Pair

### Linkage disequilibrium

**Fig. 1:**
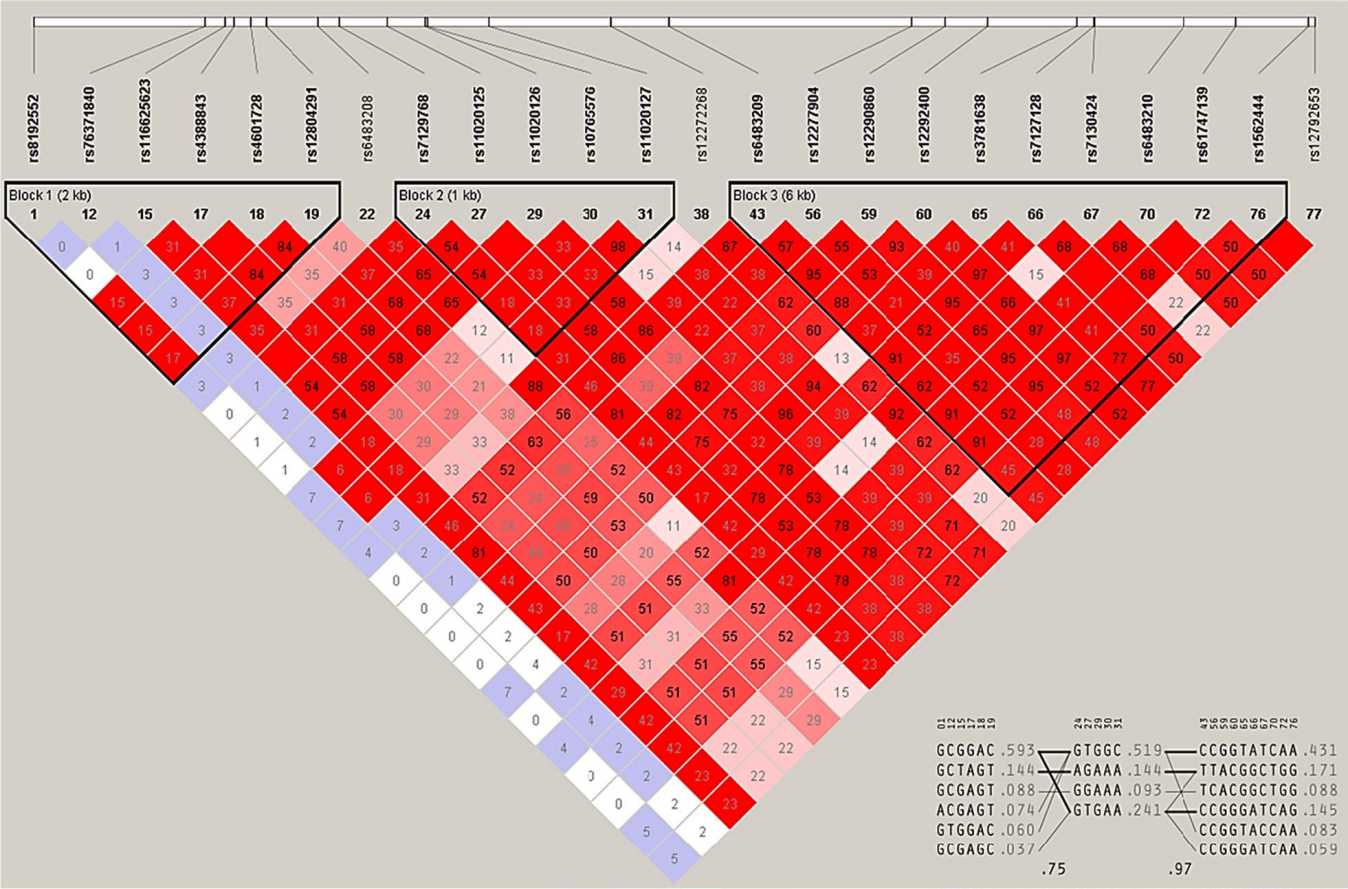
Linkage Disequilibrium Plot for SNPs in the *MTNR1B* gene. The value in each diamond is r^2^ between pairs of SNPs. The haploview standard color scheme, for LD color display with bright red (LOD ≥ 2 and D′ = 1), shades of pink/red (LOD ≥ 2 and D′ < 1), white (LOD < 2 and D′ < 1), and blue (LOD < 2 and D′ = 1). Haploview generated haplotypes blocks for SNPs in strong LD

To understand the linkage disequilibrium (LD) around the suggestively or nominally significant *MTNR1B* variants, we generated an LD plot (Fig. 1). Three haplotypes were identified from the LD plot in the *MTNR1B* genes from the JHS cohort (Fig. 1). In haplotype block 1, all the variants showed a strong LD (D′ > 1) except for rs76371840. For block 2, all the variants were in strong LD. In haplotype block 3, most variants were in strong LD except rs7130424 & rs37816638 and rs7130424 & rs1562444 (D′ < 1).

## Discussion

In this study, we examined the associations of polymorphisms in *MTNR1B* with obesity traits in a sample of African Americans at JHS. Our principal findings revealed a significant association of rs8192552 with high waist circumference even after adjusting for multiple testing. Furthermore, the T allele of rs76371840 was associated with adiposity even after adjusting for insomnia in the regression model. We also observed four new variants (allele G of rs6483208, allele A of rs4388843, allele G of rs4601728, and allele T of rs12804291) that showed an effect on adiposity outcome after adjusting for insomnia. To the best of our knowledge, we are the first to report this association.

Our results indicated a significant association of rs8192552 with high waist circumference. The variant rs8192552 is a missense variant that has been extensively studied in T2D and shows no association to T2D in European populations [19] and African American populations [20]. However, rs8192552 is significantly associated with BMI and waist circumference in French and Danish populations [19]. Elsewhere, rs8192552 did not show a significant association with BMI, as well as waist circumference [20, 21]. Andersson et al. [19] reported that the missense variant rs61747139 did not display a significant association with BMI and waist circumference. Allele G of rs61747139 has been reported to cause an amino acid change of lysine to arginine. This codon change could affect gene expression, leading to a defect in melatonin signaling pathways. Karamitri et al. [12] observed that rs61747139 decreased β-arrestin-2 recruitment, while rs8192552 did not. The β-arrestins desensitize G-protein-coupled receptors (e.g., *MTNR1B*) to prevent further stimulation of G proteins and the downstream signaling pathways [22]. The overstimulation of melatonin signaling by variant rs61747139 via β-arrestin-2 could lead to a downstream effect on markers involved in obesity.

The pairwise variants rs12792653 and rs1562444 are associated with both BMI and waist circumference. These SNPs (rs12792653 and rs1562444) are in the 3’-UTR region of the mRNA that follows the stop codon, which contains regulatory regions essential for post-transcription regulation [23]. It also contains binding sites for regulatory elements, such as microRNAs, repressors proteins, and proteins that bind to AU-rich elements (ARE-BP), which are involved in either translation activation or repression [23, 24]. Thus, 3’-UTRs act as cis-regulators.

Additionally, insomnia modified the association between the *MTNR1B* variants and adiposity because we observed four novel variants (rs6483208, rs4388843, rs4601728, and rs12804291) with a significant association with adiposity when we adjusted for insomnia. To date, few studies have investigated the association between the circadian-related gene variants with metabolic parameters [25–28]. Ollila et al. described the effect of insomnia on the association of *MTNR1B* variants on blood glucose [25]. Another study discussed the effect of diet and sleep on the association between circadian-related gene variants (*MTNR1B, CLOCK*, *CRY*, and *NR1D1*) and metabolic traits (fasting glucose, BMI, waist circumference, and HDL-cholesterol) [28]. Our study demonstrated that insomnia affected the association between *MTNR1B* variants and adiposity in an African American sample population.

While these findings are insightful to the role of *MTNR1B* variants in African Americans, a few limitations are worth mentioning. Our main limitation is the small sample size relative to studies of different ethnic backgrounds. Our study needs to be replicated in a larger African American cohort. Another limitation is the use of BMI to assess adiposity instead of the overall body fat mass. Despite these limitations, our data produced associations that were similar to other studies.

## Conclusions

In summary, we found a novel association between allele A of rs8192552 and high waist circumference. Also, allele T of rs76371840 showed an association with adiposity. Moreover, allele G of rs12792653 and allele G of rs1562444 in the 3’-UTR were associated with BMI and waist circumference. Additionally, when the data was adjusted for insomnia in the adiposity model, allele G of rs6483208, allele A of rs4388843, allele G of rs4601728, and allele T of rs12804291 showed a significant association with adiposity. In conclusion, our study identified several *MTNR1B* variants associated with obesity in the Jackson Heart Study Population. These findings contribute to understanding the link between circadian disruption (insomnia) and metabolic homeostasis.

## Methods

### Study subject

For this study, we used cross-sectional data from the Jackson Heart Study (JHS). The JHS is a single-site community-based cohort study of risk factors for cardiovascular disease among adult African American men and women living in the Jackson, Mississippi, Metropolitan area. The study participants consisted of 5,306 individuals recruited, interviewed, and examined by certified technicians for the first exam (2000–2004) [29, 30] and were followed up in 2 subsequent exams from 2005 to 2008 and 2009 to 2013. The clinic visits encompassed a physical examination, blood and urine collection, anthropometry, and data collection regarding family history, behavioral risk factors, and sociodemographics. There were 3027 participants (Mean age of 55.13 ± 12.84 and 1871 women) who consented to the genetic analysis, and their DNA samples were genotyped in the candidate gene association resource (CARe) consortium using the Affymetrix 6.0 platform. They were later imputed to 1000 genomes phase 1 [31–33]. The study was approved by the Institutional Review Board (IRB) of the National Institutes of Health. The IRB approved the participating institutions' protocol (University of Mississippi Medical Center, Jackson State University, and Tougaloo College).

### Study variables

#### Outcome variables

Our primary outcome in this study is adiposity (BMI and waist circumference). Adiposity was measured in exam visit 1, and we defined it as a BMI greater than 30 kg/m^2^ and a waist circumference greater than 102 cm and 88 cm for men and women, respectively. All the above clinical parameters were measured according to standard laboratory and clinical techniques [30].

#### Independent variables: SNP selection genotyping and imputation

All 3027 JHS samples were genotyped on the Affymetrix 6.0 based on manufacturer protocol [33]. The candidate gene approach was used to select our genetic variants from the entire set of common genetic variants in the *MTNR1B* gene located on chromosome 11q14.3 and hg19 position base-pair ordinates chr11:92,702,789–92,718,2 (plus-strand orientation). The JHS coordination centers performed the SNPs quality control, and the variants that passed were imputed with 1000G phase 1 using Cosmopolitan reference panel including all races—version 2010–11 data freeze, 2012–03-04 haplotypes [32, 33]. The imputation was completed using Minimac3 on the Michigan Imputation Server [34]; details regarding the reference panel can be found in the 1000 Genomes Project Consortium 2010 [35]. Imputed SNPs were filtered for minor allele frequency ≥ 1%, call rate ≥ 90%, HWE *p* value > 10–6, as well as the exclusion of sites with invalid or mismatched alleles for the reference panel [32]. For this study, 109 SNPs were genotyped and imputed; we focused on common variants with a minor allele frequency (MAF) ≥ 5%, and with imputation quality ≥ 80%, 30 common variants were selected for downstream analyses. Covariates were age, gender, and 10 principal components to adjust for population stratification due to admixture [17]. In additional analyses, we also adjusted for insomnia covariate. The participants were asked if they have insomnia with the answer option of “Yes,” “No,” and “Don’t Know.” Insomnia is clinically defined as the difficulty of falling and staying asleep [36].

### Statistical analysis

#### Descriptive statistics

The study variables were summarized using the mean and standard deviation (SD) for the continuous variables and proportions for the categorical variables. The continuous variables were first assessed for normality, and then were log-transformed if they were not normally distributed. The analyses for the descriptive statistics were performed using the statistical software SAS® 9.4 [37].

#### Regression analysis

Multivariate logistic regression models were fitted to assess the associations between the dosage of the *MTNR1B* genetic variants and adiposity after adjusting for age, gender, and the 10 principal components in the adiposity model. Due to the relationship between melatonin signaling and sleep, we examined the modifying effect of insomnia (adiposity-insomnia model) on our adiposity outcome by stratifying each modifier. Multivariate linear regression models (BMI model and waist circumference model) were fitted to examine the relationships between the *MTNR1B* genetic variants and the continuous obesity outcome traits of BMI and waist circumference. The linear and logistic regression models were fitted using ProbAbel v.0.5.0 genetic analysis software [38], assuming a population-based design. Although a small subset of the JHS participants belonged to a family component, we did not adjust for family structure because previous studies have shown a minimal impact on power and the inflation of the type I error [39–42]. We used a false discovery rate (FDR) to correct for multiple testing with an adjusted *p* value threshold of 0.05. The NIH dbSNP database was used to annotate the function of the *MTNR1B* variants that displayed a significant association using the regression models [43].

The variants that were statistically significant after the FDR adjustment were used to generate a linkage disequilibrium (LD) plot. Haploview (Broad Institute, MA, USA) was used to create the LD plot, and we used the Yoruba, Nigeria population as a reference [44]. Haploview generated the haplotype blocks in the LD plot whenever 95% of the informative comparisons were in strong LD while ignoring variants with an MAF < 0.05 [45].

## Data Availability

All the data in the present study required prior approval of a manuscript proposal by the Jackson Heart Study Presentation and Publications and Sub-Committee and a signed Jackson Heart Study data use agreement; Publication ID: P0946. The Jackson Heart Study provides all the details for the data access request (https://www.jacksonheartstudy.org/Research/Study-Data/Data-Access).

## Abbreviations

BMI: Body mass index
MTNR1A: Melatonin receptor type 1 gene
MTNR1B: Melatonin receptor type 2 gene
FDR: False discovery rate
HWE: Hardy–Weinberg equilibrium
JHS: Jackson Heart Study
LD: Linkage disequilibrium
LCI: Lower confidence interval
MAF: Minor allele frequency
UCI: Upper confidence interval
SE: Standard error
SNP: Single nucleotide polymorphisms
OR: Odd ratio
T2D: Type 2 diabetes
